# *Wnt16* Overexpression in Osteoblasts Increases the Subchondral Bone Mass but has no Impact on Osteoarthritis in Young Adult Female Mice

**DOI:** 10.1007/s00223-020-00682-7

**Published:** 2020-03-05

**Authors:** Anna E. Törnqvist, Louise Grahnemo, Karin H. Nilsson, Thomas Funck-Brentano, Claes Ohlsson, Sofia Movérare-Skrtic

**Affiliations:** 1 Department of Internal Medicine and Clinical Nutrition, Centre for Bone and Arthritis Research, Institute of Medicine, Sahlgrenska Academy, University of Gothenburg, 413 45 Gothenburg, Sweden; 2 BIOSCAR, Inserm, Université de Paris, 75010 Paris, France; 3 Department of Rheumatology, AP-HP, Hopital Lariboisière, 75010 Paris, France; 4 Klin Farm Lab, Department of Internal Medicine and Clinical Nutrition, Centre for Bone and Arthritis Research, Sahlgrenska University Hospital, Vita Stråket 11, 41345 Gothenburg, Sweden

**Keywords:** Osteoarthritis, WNT16, Cartilage, Mouse model, DMM

## Abstract

Epidemiological studies have shown that high bone mineral density (BMD) is associated with an increased risk of osteoarthritis (OA), but the causality of this relationship remains unclear. Both bone mass and OA have been associated with the WNT signaling pathway in genetic studies, there is thus an interest in studying molecular partners of the WNT signaling pathway and OA. Female mice overexpressing WNT16 in osteoblasts (*Obl-Wnt16* mice) have an increased bone mass. We aimed to evaluate if the high bone mass in *Obl-Wnt16* mice leads to a more severe experimental OA development than in WT control mice. We induced experimental OA in female *Obl-Wnt16* and WT control mice by destabilizing the medial meniscus (DMM). The *Obl-Wnt16* mice displayed thicker medial and lateral subchondral bone plates as well as increased subchondral trabecular bone volume/tissue volume (BV/TV) but un-altered thickness of articular cartilage compared to WT mice. After DMM surgery, there was no difference in OA severity in the articular cartilage in the knee joint between the *Obl-Wnt16* and WT mice. Both the *Obl-Wnt16* and WT mice developed osteophytes in the DMM-operated tibia to a similar extent. We conclude that although the *Obl-Wnt16* female mice have a high subchondral bone mass due to increased WNT signaling, they do not exhibit a more severe OA phenotype than their WT controls. This demonstrates that high bone mass does not result in an increased risk of OA per se.

## Introduction

Cross-sectional and longitudinal epidemiological studies have shown that men and women with high bone mineral density (BMD) are at increased risk of osteoarthritis (OA), but the causality of this relationship remains unclear [1–4]. Both bone mass and OA have been associated with the WNT signaling pathway in genetic studies and, therefore, there is an increasing interest in studying molecular partners of the WNT signaling pathway and OA [5, 6]. Functional studies have clearly shown that WNT signaling leads to increased bone mass, while inhibiting this pathway leads to decreased bone mass [5, 7]. Although the role of WNT signaling and bone mass in OA has been studied by different genetically modified mouse models, the conclusions are still elusive.

Frizzled-related protein (*FRZB*) and Sclerostin (*SOST*) both inhibit the WNT signaling pathway. Mice with no expression of either FRZB (*Frzb*^*−/−*^ mice [6, 8, 9]) or SOST (*Sost*^*−/−*^ mice [10]) exhibit an increased cortical bone mass, but only the *Sost*^*−/−*^ mice display increased trabecular bone mass and more subchondral bone mass. Both the *Frzb*^*−/−*^ and *Sost*^*−/−*^ mice develop more severe experimental OA than their wild type (WT) controls [8–10]. Deletion of the WNT pathway antagonist secreted frizzled-related protein (SFRP)-1 in mice (*Sfrp-1*^*−/−*^ mice) leads to increased trabecular bone mass in adults but not in young adults. No difference in experimental OA phenotype was seen in young adult *Sfrp-1*^*−/−*^ mice compared to WT mice [9, 11]. Furthermore, when overexpressing the WNT signaling antagonist Dickkopf-related protein (DKK)-1 specifically in osteoblasts in mice (*Col1a1‐Dkk‐1–Tg* mice), the trabecular bone mass is decreased, also in the subchondral region, and the mice display a milder experimental OA phenotype than the WT controls [12]. In mice lacking the low-density lipoprotein receptor-related protein 5 (*Lrp5*^*−/−*^ mice), WNT signaling is inhibited and both the trabecular and cortical bone mass are decreased [13]. However, the importance of LRP5 for the development of experimental OA is inconclusive [14, 15]. *Lodewyckx *et al*.* show that *Lrp5*^*−/−*^ mice have more severe experimental OA [14], while *Shin *et al*.* show that both *Lrp5*^*−/−*^ mice and mice with a conditional deletion of *Lrp5* in cartilage (*Lrp5*^*fl/fl*^;*Col2a1-Cre* mice) have milder experimental OA [15] than the WT controls. Finally, the LRP6 induces WNT signaling and a heterozygous deletion of LRP6 in mice (*Lrp6*^*+/−*^ mice) led to reduced trabecular bone mass, as well as limb deformities and a more severe experimental OA phenotype than WT controls [13, 16]. All of the OA studies described above are performed in male mice and it is not certain that the differences in OA phenotype are also true for female mice.

Thus, the relationship between bone mass and OA severity in the mouse models with altered WNT signaling is not clear. However, most mouse models suggest that high bone mass due to more WNT signaling leads to more severe OA [6, 8–10] and that less bone mass due to less WNT signaling leads to less severe OA [12], but the latter was not seen in a model with limb deformities [13, 16]. The subchondral bone close to the knee joint is known to affect joint rigidity [17–19]. Therefore, there is a possibility that the subchondral bone closer to the joint have a higher impact on OA severity than bone further away from the joint. However, not all mouse studies mentioned above have evaluated the subchondral bone.

The *WNT16* locus has been identified as a major determinant of BMD and cortical thickness in humans [20–22]. We have previously performed functional studies in mice demonstrating that absence of WNT16 (*Wnt16*^*−/−*^ mice) results in lower cortical thickness, leading to spontaneous fractures [22]. In contrast, a transgenic mouse model that overexpresses the *Wnt16* gene under the control of the osteoblast-specific rat type I α1 procollagen (*Col1α1*) promoter (*Obl-Wnt16* mice) has an increased total body BMD, mainly caused by increased trabecular bone mass [23]. In addition, local WNT16 treatment in rat tibiae enhanced BMD, suggesting that treatments targeting the regulation of WNT16 in bone might be beneficial for patients with osteoporosis. In the present study, we have induced experimental OA, by destabilizing the medial meniscus (DMM), in female *Obl-Wnt16* mice. These mice were evaluated for osteoarthritic changes in articular cartilage and in subchondral bone. As WNT16 is a WNT signaling agonist and overexpression of WNT16 in osteoblasts leads to an increased bone mass in mice [23], we hypothesize that *Obl-Wnt16* mice will have a more severe OA phenotype than their controls. Thus, this study further evaluates if high bone mass due to WNT signaling leads to more severe OA.

## Materials and Methods

### Animals

The mouse model used in this study, with osteoblast-specific WNT16 overexpression (*Obl-Wnt16*), was generated as described previously (C57BL/6 background) [23]. Briefly, we developed a transgenic mouse model with osteoblast-specific WNT16 overexpression (*Obl-Wnt16*) under the control of a 2.3-kb fragment of the rat type I α 1 procollagen promoter. The *Obl-Wnt16* mice display a specific and high overexpression of *Wnt16* in bone [23]. All experiments were carried out on female mice born from crossing a male *Obl-Wnt16* mouse with a female C57BL/6 N mouse. WT littermates were used as controls. There are no anatomical differences in the limbs of the transgenic *Obl-Wnt16* mice as compared to WT mice. Female mice were chosen in this study since we have previously shown that the female *Obl-Wnt16* mice have an increased trabecular bone mass compared to WT mice [23]. The mice were housed together in a standard animal facility under controlled temperature (22 °C) and photo periods (12 h of light, 12 h of dark) with free access to water and food pellets (B&K Universal, Sollentuna, Sweden). Animal care was in accordance with institutional guidelines. All applicable international, national, and institutional guidelines for the care and use of animals were followed. All procedures performed were approved by the ethics committee at the University of Gothenburg.

### Surgically Induced Osteoarthritis

Experimental OA was induced in female *Obl-Wnt16* and their WT controls at eight weeks of age (*N* = 15). This was done by surgically destabilizing the medial meniscus (DMM) in the right knee essentially as described by Glasson et al. [24]. In brief, the fur surrounding the knee joint was shaved and a skin incision was made over the medial aspect of the knee joint under inhalation anesthesia with Isoflurane (Forene; Abbot Scandinavia, Solna, Sweden). The joint cavity was opened, the medial meniscus and the medial meniscotibial ligament were identified, and thereafter the medial meniscotibial ligament was sectioned using a microsurgical knife. Care was taken to avoid damaging other ligaments and cartilage. The skin was then sutured. The left knee was left intact and used as control; we refer to these as ‘un-operated controls’. All mice received the analgesic Vetergesic® vet (0.3 mg/ml, Patheon UK Limited, Swindon, UK) at the time of surgery. The mice were carefully assessed for adverse events the first three days after surgery and then every week throughout the experiment. The mice were kept for eight weeks post-operatively and then sacrificed at 16 weeks of age. The lower limbs were dissected and skin was removed. The limbs were fixed for 48 h in 4% formaldehyde and then kept in ethanol (70% v/v) until further use.

### Histological Analysis

Histological analysis was performed on fixed, decalcified whole knee joints. The joints were then processed and embedded in paraffin wax according to standard techniques. Five micrometers coronal joint sections were collected at 80 μm intervals through the entire joint of all samples. Sections were then stained with Toluidine blue according to standard techniques. Histopathologic evaluation of the severity of OA was performed by an observer (AET) blinded to genotype according to the Osteoarthritis Research Society International (OARSI) recommendation of a 0–6 subjective scoring system [25]. In this classification system 0 represents normal cartilage; 0.5: loss of Toluidine blue staining without structural changes; 1: small fibrillations without loss of cartilage; 2: vertical clefts below the superficial layer and some loss of surface lamina; 3: vertical clefts/erosion to the calcified cartilage extending to < 25% of the articular surface; 4: vertical clefts/erosion to the calcified cartilage extending to 25–50% of the articular surface; 5: vertical clefts/erosion to the calcified cartilage extending to 50–75% of the articular surface; 6: vertical clefts/erosion to the calcified cartilage extending > 75% of the articular surface [25]. The 0–6 subjective OA scoring system was applied to all four regions of the joint; the medial tibial plateau (MTP), medial femoral condyle (MFC), lateral tibial plateau (LTP) and lateral femoral condyle (LFC). For a specific joint, all sections were scored but only the maximum OARSI score from each of the four regions from that joint was used in the calculations. Thus, the OA severity is expressed as the maximal score for each of the four regions or the summed maximal scores for the medial (MTP + MFC) or lateral compartment (LTP + LFC). In order to test reproducibility, a second observer (LG) also blindly assessed a randomly selected sample of joints. Three joints from each of the four groups were chosen for this purpose, accounting for 20% of the samples from the DMM experiment. Due to technical difficulties with the sectioning, *N* = 13 instead of 15 for the LFC in the WT mice, and *N* = 11 instead of 15 for the LFC and *N*  = 14 instead of 15 for the LTP in the *Obl-Wnt16* mice.

**Fig. 1:**
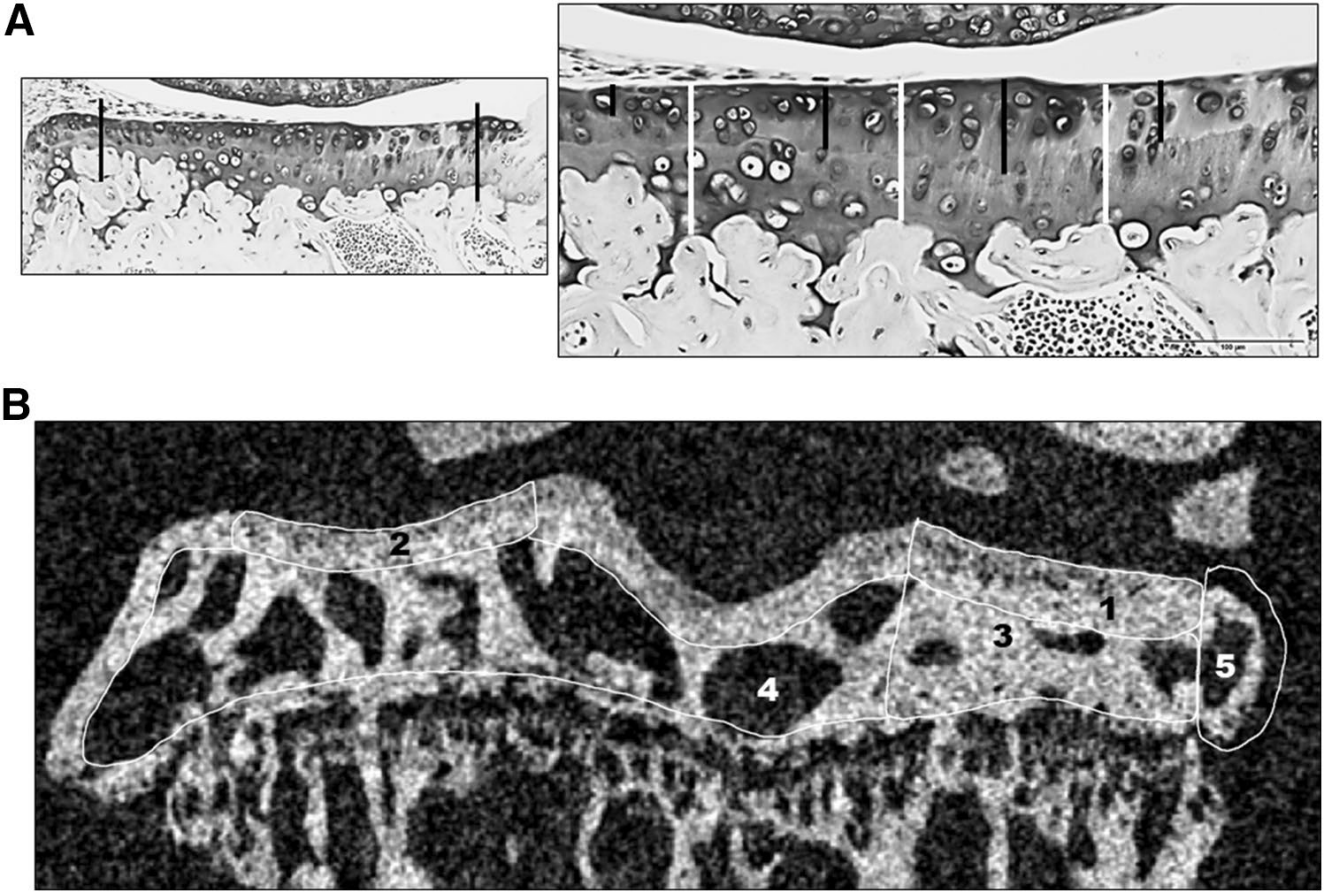
Measurements of articular cartilage and subchondral bone in tibia. **a** Photos of the articular cartilage. The left photo shows two black lines between which the measurements were made (magnification × 10). The right photo (magnification × 20, black scale bar 100 µm) shows how the measurements of the un-calcified cartilage (black lines) and the total cartilage thickness (white lines) were made. **b** Image in the coronal plane of a right DMM-operated tibia from the µCT. The regions of interest are the medial subchondral bone plate (1), the lateral subchondral bone plate (2), the medial subchondral trabecular bone (3), the total subchondral trabecular bone (3 + 4), and the osteophyte (5)

The articular thickness of the medial tibia was measured using the OsteoMeasure histomorphometry system, software version 2.2 (OsteoMetric, Atlanta, GA). The un-calcified cartilage was defined as the distance from the articular surface to the tide mark, the total articular cartilage as the distance from the articular surface to the cement line and the calcified cartilage was calculated as the total minus the calcified cartilage thickness (Fig. 1a). Twelve measurements were taken evenly distributed across the measurement area of one section per mouse and the mean from the twelve measurements was used for each mouse (Fig. 1a). *N* = 14 instead of 15 for both the *Obl-Wnt16* and their WT controls due to folding of the sections in the area where the thickness of the articular cartilage should have been measured.

### Analysis of Subchondral Bone by Micro-computed Tomography (µCT)

Analysis of subchondral bone was performed by µCT using a Skyscan 1172 model micro-CT (Bruker micro-CT, Aartselaar, Belgium) [22] with an X-ray tube voltage of 50 kV, a current of 201 µA and with a 0.5-mm aluminum filter. The scanning angular rotation was 180°, and the angular increment was 0.70°. The voxel size was 4.75 µm isotropically. The images were reconstructed using the Skyscan NRecon software and analyzed using Skyscan CTAn software. The regions of interest were the medial and total subchondral trabecular bone situated within the tibial epiphysis, the subchondral bone plates of the medial and lateral tibial plateaus and the osteophytes formed on the medial part of the tibia in the knees of the DMM-operated mice (Fig. 1b). The analyses were performed in the coronal plane.

### Statistical Analysis

Data are presented as scatter plots with the bar indicating the arithmetic mean or expressed as mean ± standard error of the mean (SEM). For comparison between two groups, Student’s *t* test was used. To evaluate the effect of surgery (within-subject effects), genotype (between-subject effects), and interaction (surgery by genotype) for the dependent variable, a mixed-design two-way repeated-measures ANOVA was used. Effect sizes (ES) are given as partial eta squared from the ANOVA. In all cases, *P* < 0.05 was considered statistically significant. The sample size for the DMM experiment was chosen to provide at least 95% power to detect a 1.6 standard deviation difference in OA severity. To confirm the inter-rater reliability of OARSI scoring we calculated the intraclass correlation coefficient (ICC) between the OARSI scores of AET and LG using reliability analysis with a two-way-random model and absolute agreement in IBM SPSS Statistics for Windows, Version 25.0 (Armonk, NY: IBM Corp.) [26].

## Results

### Reproducibility of the Scoring System

The reproducibility of the OARSI scoring system used to assess OA severity in all four joint regions in the DMM-operated and un-operated control knees was evaluated by two observers (AET and LG). AET independently and blindly assessed all knees and LG assessed 20% of the knees to analyze the inter-rater reliability. This showed an intraclass correlation coefficient of 0.940 (95% confidence interval: 0.926, 0.951) which is considered excellent.

### Role of WNT16 Overexpression by Osteoblasts in the Development of Experimental OA in Female Mice

#### Subchondral Bone

**Fig. 2:**
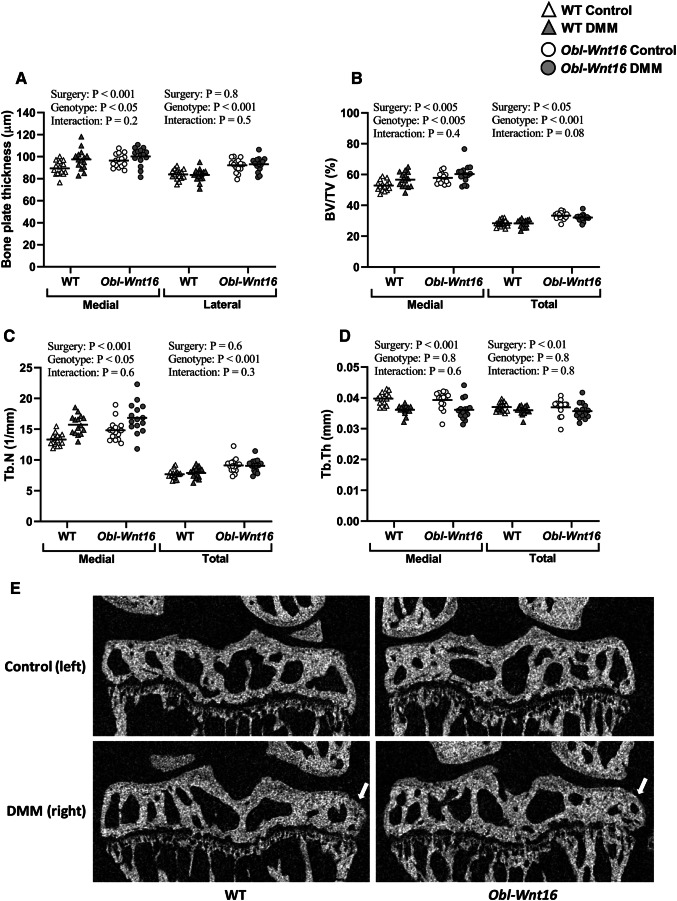
The subchondral bone. A mixed-design two-way repeated-measures ANOVA was used to assess the effects of genotype (WT vs. *Obl-Wnt16*) and surgery (un-operated [Ctrl] vs. surgery destabilizing the medial meniscus [DMM]) on the subchondral bone. **a** The subchondral bone plate thickness in the medial and lateral tibia was affected by the genotype, where *Obl-Wnt16* mice had thicker bone plates than the WT mice. The DMM surgery had an effect on the medial, but not the lateral, subchondral bone plate, where the surgery led to increased bone plate thickness. **b** The medial and total subchondral bone volume/tissue volume (BV/TV) was affected both by genotype and by surgery. The *Obl-Wnt16* mice had higher BV/TV than WT mice and the surgery led to increased BV/TV. **c** The medial subchondral trabecular number (Tb.N) was affected both by genotype and by surgery, whereas the total subchondral Tb.N was only affected by genotype. The *Obl-Wnt16* mice had a higher medial and total subchondral Tb.N than the WT mice and the DMM surgery led to a higher Tb.N on the medial side. **d** The medial and total subchondral trabecular thickness (Tb.Th) was decreased after DMM surgery, while the genotype had no effect. **e** Micro-CT reconstructions show subchondral bone, mainly in the tibia, of the left un-operated control knees (control) and the right knees that underwent DMM surgery. The arrows point at osteophytes. Data are shown as scatter plots where the bars show the mean (*N* = 15/group)

The tibial bone plate thickness on both the medial and lateral side of the knee was affected by the genotype (medial ES = 14%, lateral ES = 45%), where the *Obl-Wnt16* female mice have thicker bone plates compared to WT mice (Figs. 1b and 2a). As expected, only the medial bone plate thickness is affected by the DMM surgery (ES = 33%). Significant genotype by surgery interactions were not detected for the medial or lateral bone plate thickness (Fig. 2a). In addition, both the medial and total subchondral trabecular BV/TV (medial ES = 26%, total ES = 53%) and trabecular number (medial ES = 17%, total ES = 36%) were affected by the genotype, showing an increase in *Obl-Wnt16* female mice compared to WT mice (Fig. 2b, c). The DMM surgery also increased the medial and total subchondral trabecular BV/TV (medial ES = 28%, total ES = 14%) and the medial trabecular number (ES = 58%). There was no significant genotype by surgery interactions for the medial and total BV/TV or trabecular number (Fig. 2b, c). The subchondral trabecular thickness was not affected by the genotype but was decreased by the DMM surgery (medial ES = 61%, total ES = 24%). A significant genotype by surgery interaction was not detected for the subchondral trabecular thickness (Fig. 2d). These results are consistent with what has been shown in our previous study, where the trabecular bone mass in both vertebra and the distal metaphyseal region of femur was increased in the *Obl-Wnt16* mice compared to WT mice [23]. Osteophytes were developed to a similar extent in DMM-operated knees in both *Obl-Wnt16* and WT mice (Table 1, Figs. 1b and 2e). In the present study, there was no difference in body weight between the *Obl-Wnt16* and WT mice at the start (data not shown) or at the termination of the experiment (*Obl-Wnt16*: 22.7 ± 0.4; WT 23.6 ± 0.6 g, *P* = 0.19).

**Table 1:** Osteophyte volume in the tibia of DMM-operated knees and thickness of medial articular cartilage in un-operated control knees from WT and *Obl-Wnt16* mice

	WT	*Obl-Wnt16*
Osteophyte volume (mm^3^)	0.059 ± 0.005	0.056 ± 0.005
Total articular cartilage thk (μm)	83.8 ± 2.0	83.9 ± 1.8
Un-calcified articular cartilage thk (μm)	39.0 ± 1.6	38.1 ± 1.9
Calcified articular cartilage thk (μm)	44.8 ± 2.4	45.7 ± 2.4

Data are presented as means ± SEM (osteophyte volume: *N* = 15, articular cartilage thickness: *N* = 14). There were no significant differences between the WT and *Obl-Wnt16* mice in osteophyte volume or any measures of the articular cartilage thickness. The statistical analyses were performed using Student's *t* test

*Thk* thickness

#### Articular Cartilage

There was no difference in articular cartilage thickness (total, un-calcified, and calcified) on the medial side of the un-operated knees comparing the *Obl-Wnt16* and the WT mice (Table 1).

**Fig. 3:**
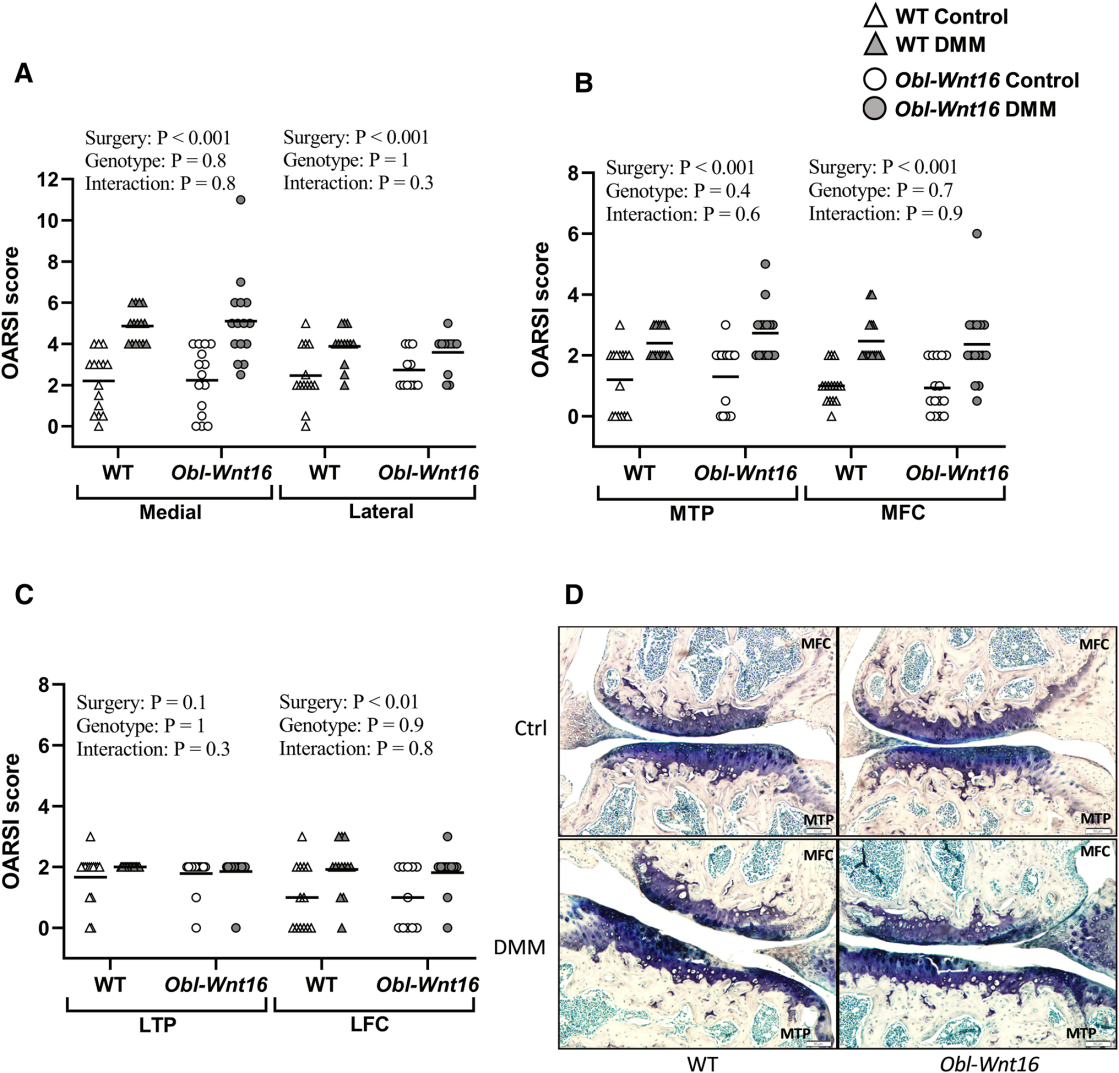
OARSI scores for un-operated control knees and knees that underwent DMM surgery. A mixed-design two-way repeated-measures ANOVA was used to assess the effects of genotype (WT vs. *Obl-Wnt16*) and surgery (un-operated [Ctrl] vs. surgery destabilizing the medial meniscus [DMM]) on OARSI scores. **a** The summed OARSI scores for each compartment (medial = medial tibial plateau [MTP] + medial femoral condyle [MFC]; lateral = lateral tibial plateau [LTP] + lateral femoral condyle [LFC]), where the maximum score for each compartment is 12. The surgery but not the genotype affected the summed medial and lateral OARSI score. **b** The surgery but not the genotype affected the OARSI score in the MTP and in the MFC. The maximum score for each region is 6. **c** The surgery but not the genotype affected the OARSI score in the LFC. The maximum score for each region is 6. **d** Representative microphotographs of the MTP and MFC in un-operated control (Ctrl) and DMM-operated knees in *Obl-Wnt16* and WT mice. Especially the MTP in the DMM-operated knees show severe cartilage damage both in the *Obl-Wnt16* and WT mice. Data are shown as scatter plots where the bars show the mean (summed lateral compartment and LFC in *Obl-Wnt16* mice *N* = 11; LTP in *Obl-Wnt16* mice *N* = 14; summed lateral compartment and LFC in WT mice *N* = 13; all other parameters *N* = 15/group)

First, the maximum OARSI scores for the medial (MTP + MFC) and lateral compartments (LTP + LFC) were summed. Both the summed medial and lateral compartments (medial ES = 66%, lateral ES = 43%) had higher OARSI scores after DMM surgery than the un-operated control knees, but this was not affected by the genotype (Fig. 3a, d). Significant genotype by surgery interactions were not detected for the summed OARSI scores (Fig. 3a). Since the summed OARSI scores for both the medial and lateral compartment were significantly altered by surgery, all four regions of the articular cartilage were analyzed separately. The OARSI scores for the MTP and MFC were affected by the surgery (MTP ES = 58%, MFC ES = 57%) but not the genotype (Fig. 3b, d). On the lateral side, only the LFC was affected by the surgery (ES = 29%) and the genotype showed no effect on the LTP or LFC (Fig. 3c). Significant genotype by surgery interactions were not detected for the OARSI scores in any of the four regions (Fig. 3b, c).

## Discussion

Epidemiological studies have shown that high BMD is associated with a high risk of OA. When longitudinal epidemiological studies were evaluated, the results mainly suggested an association between the incidence of OA and high BMD but no association between OA progression and high BMD [1, 3, 4, 27, 28]. WNT signaling is a major regulator of bone tissue and genetic studies have found several candidate genes from the WNT signaling pathway that are associated with OA [6]. Therefore, there has been a major interest in evaluating how WNT signaling is associated with OA development. Herein, we have shown that a high subchondral bone mass, due to overexpression of WNT16 in osteoblasts, does not lead to a more severe OA phenotype in female mice after inducing experimental OA by DMM surgery.

Some previous mouse studies, where mice had a deletion or overexpression of a gene involved in WNT signaling, showed a correlation between high bone mass and more severe OA (*Frzb*^*−/−*^ mice [8, 9] and *Sost*^*−/−*^ mice [10]) and between low bone mass and less severe OA (*Col1a1‐Dkk‐1–Tg* mice [12]). However, this was not seen for the *Lrp6*^*+/−*^ mice, which had a reduced trabecular bone mass and displayed a more severe OA compared to WT mice [16]. The limb deformities in this mouse model probably contribute to the OA severity, as secondary OA often can be seen in individuals with limb deformities caused by e.g., Paget’s disease of bone [29]. Experimental OA was studied in the *Sfrp1*^−/−^ mice when they were young adults and the trabecular bone mass was not increased at that age [9, 11]. One may speculate that the lack of a more severe experimental OA in these mice is due to a normal trabecular bone mass at the time of the study and it is also possible that the subchondral bone mass is not affected in the *Sfrp1*^−/−^ mice. In order to further evaluate the relationship between bone mass, WNT signaling, and OA we have induced experimental OA by DMM in female *Obl-Wnt16* mice which overexpress the WNT signaling agonist WNT16 in osteoblasts [23]. Although the *Obl-Wnt16* mice had an increased trabecular bone mass in the subchondral compartment and thicker subchondral bone plates, as well as increased trabecular bone in the metaphysis of the femur as compared to WT mice, they did not differ from the WT mice in OA severity. Together with results from previous mouse studies on low bone mass due to inhibition of WNT signaling (in the *Col1a1‐Dkk‐1–Tg* mouse model), or high bone mass due to increased WNT signaling (in the *Frzb*^*−/−*^ and *Sost*^*−/−*^ mouse models), our data on high bone mass gained by increased WNT signaling suggest that the bone mass per se is not the only contributor to the OA phenotypes in these mice, but that other mechanisms are probably also important. There is also a possibility that there is a threshold in bone mass, above which it is more likely that the mice develop OA traits and that the increased subchondral bone mass in the *Obl-Wnt16* mice is not above this threshold.

The *Obl-Wnt16* mice have an osteoblast-specific *Wnt16* overexpression under the control of a 2.3-kb fragment of the rat type I α 1 procollagen promoter. It has previously been shown that activation of this promoter fragment leads to expression specifically in osteoblasts and osteocytes with no detectable expression in chondrocytes [30–32], although we did not perform these analyses in our mice. In addition, our previous publication shows that the *Obl-Wnt16* mice have a specific overexpression of WNT16 in bone and not in uterus, as there was no change in the expression of *Wnt16* mRNA in uterus, whereas there was a 140-fold increase of *Wnt16* mRNA in trabecular bone and a ninefold increase in cortical bone compared to WT mice [23].

We have previously shown that *Wnt16*^*−/−*^ mice, on a C57BL/6 N genetic background, display lower cortical thickness leading to spontaneous fractures [22]. Due to the development of spontaneous fractures, this *Wnt16*^*−/−*^ mouse model cannot be used for experimental OA studies. However, *Wnt16*^*−/−*^ mice on a 129/Sv genetic background have no incidence of spontaneous fractures [33]. These *Wnt16* deleted 129/Sv mice developed more severe OA after DMM surgery compared to control mice [33]. Furthermore, mice with cell-specific deletion of *Wnt16* in chondrocytes (*Col2α1-creWnt16*^flox/flox^ mice) also developed more severe experimental OA than controls, whereas their bone phenotype was normal [34].

The direct impact of WNT16 for the development of OA has been further investigated by use of adenoviral *Wnt16* vectors, overexpressing *Wnt16*. Adenoviral *Wnt16* vectors injected intra-articularly after experimentally induced OA, attenuated the OA phenotype in both WT and *Col2α1-creWnt16*^flox/flox^ mice [34]. However, another study showed that mice not undergoing any procedure to develop experimental OA displayed erosive lesions in the articular cartilage after intra-articular injection of an adenoviral *Wnt16* vector, due to an increase in canonical WNT signaling [35]. These data suggest that WNT16 exerts positive effects on cartilage during an ongoing OA but in healthy joints, WNT16 exerts negative effects on the cartilage.

Epidemiological studies have shown that high BMD is associated with a high risk of OA and the longitudinal epidemiological studies specifically showed that there were associations between incidence of OA and high BMD, whereas most studies found no association between high BMD and OA progression [1, 3, 4, 27, 28, 36]. In our *Obl-Wnt16* mice, there was no difference in OA severity after the DMM surgery compared to WT mice. The DMM surgery is an operation in the knee, cutting the medial meniscus, which causes OA in normal WT mice. Our hypothesis was that the high bone mass in *Obl-Wnt16* mice would lead to a more severe OA phenotype eight weeks after surgery. In the present study, we did not see a more severe OA in the *Obl-Wnt16* mice compared to controls, which would be in line with what has been seen in epidemiological studies where the progression of the disease was not associated with high bone mass. One limitation of the present study is that only young adult and not older mice were studied. It would also be interesting to evaluate whether mice with a high bone mass may have an increased risk of developing spontaneous OA at a higher age. Another limitation with the study is that the effect of WNT16 overexpression on the subchondral bone mass and OA was only evaluated in female mice. Although female mice develop a less severe OA after DMM surgery than male mice [37] we herein show that the females do develop a mild OA. Our hypothesis was that the *Obl-Wnt16* mice would have a more severe phenotype after DMM surgery than the control mice, this would have been detected using this model. Further studies are necessary to investigate whether the results also apply in male mice.

In conclusion, the high subchondral bone mass in the *Obl-Wnt16* mice, due to increased WNT signaling specifically in bone, does not lead to a more severe OA phenotype after DMM surgery compared to WT mice. Thereby, our results suggest that treatment of osteoporotic patients with strategies targeting the regulation of WNT16 would probably not lead to an increased risk of developing OA. We propose that bone mass is not the main contributor to OA severity and that there are other mechanisms also involved in experimental OA development in mice.

## Data Availability

The data that support the findings of this study are available from the corresponding author upon reasonable request.
